# The Shisong Cardiac Center in Cameroon: An Example of a Long-Term Collaboration/Cooperation Toward Autonomy

**DOI:** 10.3389/fped.2018.00188

**Published:** 2018-07-03

**Authors:** Alessandro Giamberti, Gianfranco Butera, Charles MVE Mvondo, Silvia Cirri, Alessandro Varrica, Nadia Moussaidi, Giuseppe Isgrò, Jean Claude Ambassa, Cabral Tantchou, Giovanni Giamberti, Alessandro Frigiola

**Affiliations:** ^1^Department of Pediatric Cardiology and Cardiac Surgery, IRCCS Policlinico San Donato, San Donato Milanese, Italy; ^2^Association “Bambini Cardiopatici nel Mondo” ONG, Milan, Italy; ^3^Cardiac Center Shisong, Kumbo, Cameroon; ^4^International Cooperation Unit, IRCCS Policlinico San Donato, San Donato Milanese, Italy; ^5^Department of Anesthesiology and Intensive Care, IRCCS Policlinico San Donato, Milan, Italy; ^6^Purchase Department, IRCCS Policlinico San Donato, San Donato Milanese, Italy

**Keywords:** health, cooperation, cardiac surgery, congenital heart disease, collaboration project

## Abstract

Congenital heart diseases (CHD) are present in nearly 1% of live births; according to WHO, there are 1. 5 million newborns affected by CHD per year and more than 4 million children waiting for cardiac surgery treatment worldwide. The majority of these children (~90%) could be treated, saved and subsequently have a good quality of life but unfortunately, in developing countries with a suboptimal care or no access to care, they are destined to die. Cameroon, one of the 40 poorest countries in the world, is a typical example of this dramatic scenario and this is why we started a collaboration project with a local religious partner (Tertiary Sisters of Saint Francis) in 2001 with the aim of establishing the first cardiac surgery center in this country. There are various well-known organizational models to start a cooperation project in pediatric cardiac surgery in a developing country. In our case, the project included a long-term collaboration with a stable local partner, a big financial investment and a long period of development (10 years or more). It is probably the most difficult model but it is the only one with the greatest guarantee of success in terms of sustainability and autonomy. The aim of this study is to analyze the constructive and problematic aspects of the 17-year collaboration in this project, and to assess possible solutions regarding its critical issues. Although much has been done during this 17-year we are aware that there is still a lot that needs to be done.

## Introduction to the problem

Cardiovascular diseases are the leading cause of death worldwide and, despite the progress of medicine in the last 25 years in this field, 17.5 million deaths annually occur due to these non-communicable diseases (1).

Congenital heart diseases (CHD) are present in nearly 1% of live births, according to WHO there are 1.5 million newborns per year affected by CHD and more than 4 million children waiting for cardiac surgery treatment in the world (2).

The majority of these children (~90%) could be treated, saved and subsequently have a good quality of life. Unfortunately, children from developing countries are destined to die because they receive suboptimal care or have no access to healthcare (3). There is a dramatic disproportion between medical access and healthcare services within developed and developing countries, 89% of the world's healthcare resources are used only for 7% of the sick people (World-Health-Statistics, 2015). The unequal distribution and access to healthcare facilities are particularly evident when it comes to cardiac surgery. Data from literature demonstrate that there is a cardiac surgery center in the US every 120,000 people, in Australia and Europe each center can provide healthcare for 1,000,000 people. In Asia the number of people treated per center increases up to 16,000,000, the worst situation is seen in Africa with a rate of 1 center every 33,000,000 people (4, 5). If we analyze data regarding the CHD incidence (6), Novick and Cardarelli (7) estimate that 810,000 children born with moderate to severe forms of CHD should be surgically treated every year but <1.5% of them outside the industrialized countries receive the necessary surgical care (8).

Cameroon, one of the 40 poorest countries in the world, is a typical example of this dramatic scenario and this is the reason why we started a collaboration project with a local religious partner (Tertiary Sisters of Saint Francis, TSSF) in 2001, with the aim of establishing the first cardiac surgery center in the country.

## Cameroon

The Republic of Cameroon is situated in Central Africa at the juncture of the Gulf of Guinea with an estimated population of 23,920,887 in 2016. French and English are the official languages which are spoken by 70 and 30% of the population, respectively. There are two international airports served by the most important air companies in Yaoundè (administrative capital) and in Douala (economic capital).

In Cameroon, 45% of the population is under 15 years of age, as expressed in Table 1 the birth rate of Cameroon is 36.2‰ with an infant mortality of 63‰, the life expectancy is 54.8 years for males and 57.1 years for females (9).

**Table 1:** Comparative data between Italy and Cameroon.

	**Italy**	**Cameroon**
Surface in km^2^	301,341	475,442
Population	60,000,000	24,000,000
People younger man then 15 yr	15%	45%
Birth rate	7.8‰	36.2‰
Infant mortality	2.9 ‰	63‰
Life expectation male	80.6 yr	54.8 yr
Life expectation female	85.1 yr	57.1 yr

*Calendario Atlante De Agostini 2018, Istituto Geografico De Agostini, (11/2017). p. 418–419; p.229–230*.

The Cameroon healthcare system is officially organized in three levels: the Public Sector managed by the Ministry of Health, the Private Sector generally managed by NGOs and the Traditional Medicine sub-sector. The reality is that in Cameroon, missionaries are so far the great stakeholders in the health-domain especially in the peripheral area. The TSSF, local partner of our project, have been responding to the sick people's needs in Cameroon since 1935. They operate in four hospitals and 12 health-centers across the country. The most important hospital is the St. Elisabeth Catholic General Hospital (SECGH) in Shisong/Kumbo, in the anglophone North-West Province.

This is a general hospital with 350 beds including different departments (obstetrics/gynecology, urology, general-surgery, dentistry, ophthalmology, pediatrics, radiology, and infective-disease), laboratories, pharmacy and an internal Nurse School of Health Sciences. The hospital has been officially recognized by the Cameroonian Government since 1952.

In 2000, the Administration of the SECGH contacted our NGO (*Bambini Cardiopatici nel Mondo Association*) and our Hospital (*IRCSS Policlinico-San Donato*) in order to collaborate for the realization of the first Cardiac Center (CC) of Cameroon.

## Shisong cardiac center project

After several visits, our NGO, together with another Italian NGO (Cuore Fratello), and the TSSF started the project in 2002, with the objective of establishing a center which could be completely autonomous.

We spent 8 years for the initial phase that included the building construction, staff training and instrumental equipment of the CC, it ended with the inauguration of the center on the 19th of November, 2009.

During this phase several training and diagnostic missions were carried out, 120 urgent cases of children with CHD were transferred to Italy for cardiac surgery. The total economic investment by the 3 partners consisted of about 6 million euros.

**Figure 1:**
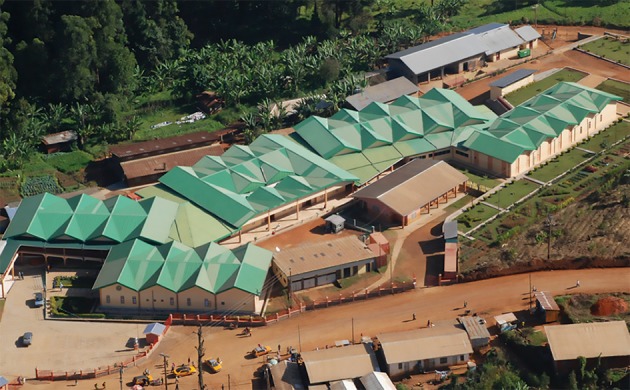
Overview of the CC: the green roofs are the Cardiac Center [Internal archive, IRCCS Policlinico San Donato, San Donato M.se, (Mi), Italy].

The CC is today a modern complex of 3,100 covered square meters (Figure 1) with 7 blocks including:

Block-A: out patients department (reception, public-relations office, pharmacy, consultation-rooms, secretariat, administration, social-case office);
Block-B: pre/post-surgical unit;
Block-C: clinical unit;
Block-D: extended wards and X-Ray unit;
Block-E: critical area with cath lab, 2 operating theaters, and 2 intensive-care units with a total of 12 fully-equipped beds;
Block-F: left basement with technical department, procurement office and drugstore;
Block-G: right basement with a conference-hall, meeting-rooms, blood-bank, guest-house, changing rooms, laundry and research-committee office.

The infrastructures created are: a water-treatment plant, 2 uninterruptible power-supply, 2 standby power generators, autonomous oxygen production central, complete laundry service, blood-bank, and more recently an autonomous incinerator plant.

During the first 8 years, 3 doctors (2 cardiologists and 1 cardiac surgeon), 15 nurses, 4 technicians, 2 perfusionists, 1 pharmacist, 1 biomedical-engineer were trained in Italy in our Hospitals. All of them are Cameroonians, they trained in our Hospitals from a minimum of 6 months to a maximum of 2 years.

The cardiologists are now independent consultants in the CC and they can also operate with a Mobile Unit in the most important cities of Cameroon (Yaoundè, Douala, Bamenda, Bafoussam, and Garoua).

A scientific research committee has been created to stimulate and support the scientific activity with publications and congress presentations. The committee includes 2 local members and 2 foreign members with expertise in cardiac surgery and cardiological research, well-recognized at an international level.

## Results

The CC is the only center performing cardiac surgery in Cameroon. In only a few years it has become a national reference center for cardiology and cardiac surgery activity for both pediatric and adult patients. The CC provides a total of 74 beds: 12 in ICU, 22 Post-Op, 20 for female hospitalization and 20 for male hospitalization. The healthcare team includes 56 nurses, 2 pharmacists and 4 physicians (2 cardiologists, 1 cardiac surgeon, and 1 anesthesiologists). In collaboration with the medical staff, there is also an administrative staff responsible for the non-medical management.

After an initial training phase, the cardiological activity at the CC is now improving every year, reporting ~6,500 cardiological consultations each year with an average of 3,000 echo and EKG procedures per year.

The invasive activity includes cardiac surgery for CHD and acquired heart disease (AHD), diagnostic and interventional catheterization, and PMK implantation (Table 2); 719 patients underwent cardiac surgery between November 2009 and December 2017, including operations for 302 CHD (42%) and 417 AHD (58%) (Table 3), with a mean of 80 patients operated per year. The most frequent operations performed are shown in Table 4. The total activity of the CC is shown in Table 5. In the last 3 years, the surgical mortality at 30 days has been of 4.7% for CHD and 4.2% for AHD patients. The local team is completely autonomous for the AHD surgery, while for CHD patients the team still needs the support from surgical mission teams from foreign countries. In 2017, 43 patients with CHD have been operated during 5 surgical missions (3 from Italy, 1 from Belgium, and 1 from Mozambique), 30 of them (70%) have been operated by the local surgeon helped by more experienced surgeons and 13 by surgeons coming from abroad.

**Table 2:** Summary of clinical activities of the Cardiac Center of Shisong from November 2009 to December 2016 (Internal database, Shisong Cardiac Center, Cameroon).

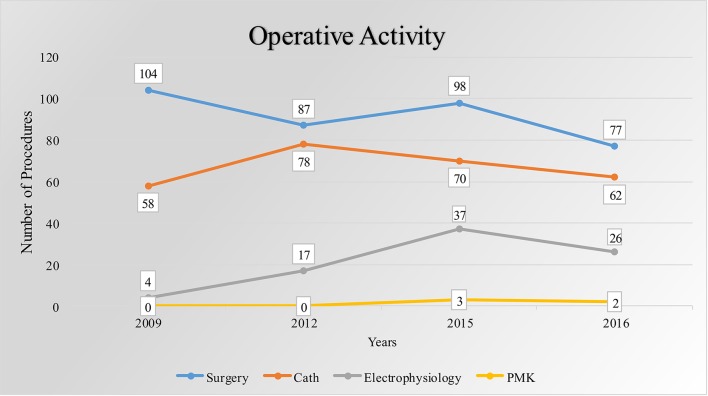

*The blue curve correspond to the number of surgeries. Similarly, the gray line indicates the electrophysiology activities*.

**Table 3:** Surgical activity of the CC (Internal database, Shisong Cardiac Center, Cameroon).

**Years**	**Congenital heart disease**	**Acquired heart disease**
2009–2015	221	332
2016	38	39
2017	43	46
Total	302	417

**Table 4:** Most frequent cardiac operations performed (Internal database, Shisong Cardiac Center, Cameroon).

**CHD (302)**			**AHD (417)**		
**Surgery**	**Nr**	**%**	**Surgery**	**Nr**	**%**
TOF	86	28.4	Mitral replacement	151	36.2
VSD	82	27.1	Aortic replacement	64	15.37
ASD	29	9.6	Mitro-aortic replacement	67	16.0
PDA	30	9.9	Mitral repair	41	9.8
AVSD	21	6.9	Thoracic aortic aneurysm	26	6.2
Others	54	17.8	CABG	17	4
			OTHERS	51	12.2

*TOF, Tetralogy of Fallot; VSD, Ventricular Septal Defect; ASD, Atrial Septal Defect; PDA, Patent Ductus Arteriosus; AVSD, Atrio-Ventricular Septal Defect; CABG, Coronary Artery Bypass Graft Surgery; PM, Pacemaker; ECG, Electrocardiogram*.

**Table 5:** Productivity enhancement since 2009.

**Activity**	**2009–2014**	**2015**	**2016**	**2017**	**Total**
Consulation	27.866	6.313	6.561	6.191	46.931
Echo diagnosis	12.475	2.956	3.063	2.632	21.126
ECG diagnosis	11.642	2.579	2.787	2.409	19.417
In-patients	5.621	1.342	1.099	1.082	9.144
Electrophysiology	87	40	28	24	179
Catheterization	309	70	62	58	499
Surgeries	455	98	77	87	717
Holter	255	70	47	81	453
Mobile consulation	6.338	3.625	3.335	3.385	16.683

The number of cardiac surgery procedures depends on the funds that various charity associations, NGOs, and Foundations make available to cover the costs of the interventions.

Most of the patients assessed and operated at the CC come from Cameroon, especially from the near provinces of Littoral, Center and North-West. In the last 2 years, patients from other countries such as Democratic Republic of Congo and Equatorial Guinea have also been operated, demonstrating that the CC is a reference center not only for the local population but also for the neighboring population.

Out of the hospital environment, it is important to highlight that the consumable goods completely purchased abroad at the beginning of the CC, are now purchased on the local market when it comes to 40% of the goods.

From a scientific point of view, the CC medical staff in collaboration with the foreign staff has produced 20 publications on Index-Medicus since 2009.

## Discussion

There are different well-known organizational models to develop a cooperation project in pediatric cardiac surgery in a developing country. Hereunder, we have synthesized the most frequent and probably the best known collaboration models.

### First

To send people in a foreign country for education and training in excellent centers for a period of variable duration. These people must come back to their country and start a local program in pediatric cardiac surgery. This model takes a long-time and frequently the training is not enough because in western countries the training stage can only be observational and not practical for legal reasons.

### Second

To have visiting teams from abroad regularly visiting an institution doing surgery and training “on-site.” Usually they spend short periods, several times a year, for a variable number of years. This is the most frequently used model and the easiest to use. It is expensive and the local training has been shown to be ineffective and suboptimal for skill transfer (9), and entirely dependent on donations.

### Third

To have a senior physician, usually a retired cardiac surgeon or cardiologist who desires to help a developing country. This model needs a governmental support and often the senior leader is not enough to develop a local autonomous program.

### Fourth

To establish a long-term collaboration between a local team/hospital and a well-known experienced international partner. This model requires stable partners, great financial investments, and a long period of development (10 years or more). It is the most difficult model to implement but the one with the greatest guarantee of success in terms of sustainability and autonomy.

The CC project can be included in this latter model of cooperation. Our NGO is involved only in collaborative projects with the goal of creating stable and autonomous cardiac surgery centers in developing countries (3). The pioneer partners of our project have remained the same since the beginning (2001); this project required a lot of time for the developing phase. The work is not finished yet because a lot of time to complete the autonomy and sustainability of the CC will be needed.

Since the beginning, we conducted this project focusing not only on the surgical aspect but also trying to develop all the intrinsic aspects of an optimal medical-care organization. We have built a center with the most modern and complete medical equipment, providing also support infrastructures like the oxygen production central and, recently, an autonomous incinerator-plant but especially we have allocated a lot of resources for local staff training. As reported, since the beginning, the CC is a Cameroonian center totally managed by Cameroonian people, but above all it is a healthcare center for Cameroonian patients.

Not only the medical and nursing personnel have attended the training program, but also all the professional figures involved in all the CC activities such as technicians, perfusionists, pharmacists, biomedical-engineers, and administrators. At the same time, these people are continuously involved in the on-site training of the younger personnel.

Complete autonomy has not yet been reached by the healthcare staff: at least 2 cardiologists, a cardiac surgeon, an anesthetist and an intensivists are still missing.

The evaluation of the surgical procedures performed and the results achieved present both positive and critical aspects. It is relevant that mortality is <5% both for CHD and AHD patients. Unfortunately, the center is not yet autonomous for the medical care of CHD and much work is still necessary to increase the number of interventions performed per year and to avoid that the CC will be underutilized. The structures and infrastructures present at the CC potentially allow to perform more than 400 operations per year but only 80 take place.

There are various explanations for this discrepancy. Certainly, the lack of autonomy of the medical personnel plays an important role, but the greatest limit is the economic one.

The number of patients operated is related to the funds that various charity associations, NGOs, and Foundations make available to cover the costs of the intervention. Very few patients are able to pay for the cost of an intervention and the government's economic support is minimal or null. There are also cultural limits. The presence of a missionary religious order as local partner is, on the one hand, a guarantee of a stable, reliable, honest and continuous partner, which has been present on site for about 100 years. On the other hand, it is a partner without business-skills but rather bound to a celestial vision that Providence will help in any case. Moreover, the missionary religious order often does not have an incisive political weight in the government decisions and a supported economic strength.

Nine years after the inauguration, we can say that the government support has been minimal, and it is difficult to think that the situation will improve in the future. When it comes to this, during these years we hypothesized various explanations: a depressed economy, absence of a true health development plan, disorganization, corruption and absence of a national healthcare system. At the same time the existence of unhealthy competition and jealousy must also be considered. Over the past 20 years, many expensive and unsuccessful attempts were made in order to open other cardiac surgery centers in Yaoundè and Doaula, all without success. Moreover, every local hospital/university wants to grow and refuse to refer patients to the CC even if their service is not readily available; this lack of collaboration can only destabilize the progress and the development of both the CC and the other cardiac centers. Table 6 summarizes the barriers and the facilitators that we identified during the set-up of the CC.

**Table 6:** Barriers and facilitators to set up the CC.

**Barriers**	•Economic limit
	•Minimal or null government support
	•Absence of true development health plan
	•Disorganization
	•Corruption
	•Absence of a true national healthcare system
	•Culture limit
	•Low incisive political weight of the local partner
	•Absence of complete local medical staff team
**Facilitators**	•Stable, reliable, honest local partner
	•Long-term collaboration between same partner
	•Great enthusiasm
	•Professional training of local people before the opening of the CC
	•Modern complex with the best instrumental equipment
	•Cameroonians employers at every level

Most importantly, our data from CC should be evaluated in the African context of the Sub-Saharan area. A recent published study (5) showed that in 2012 twenty-two centers in the Sub-Saharian area performed 1,277 open heart operations with a mean of 58 operations per year. The majority of these centers offer open heart surgery only in complete collaboration with and dependency on foreign visiting teams. In this context, the quantitative and qualitative results produced at the CC assume a value that is certainly more positive than the raw analysis of numbers.

We have recently sent a simple questionnaire with three questions: (1)what has been working in this project? (2)what was wrong (not working) with this project? (3)what do you suggest to improve in the project in the future? to 100 people who have been involved in the project since 2001 (nurses, physicians, engineers, administrative staff, volunteers).

For the first question, the most frequent answers were: great enthusiasm, ability to work in partnership, ability to think “big” and professional training before the opening of the CC. For the second question, the answers were: western/global economic crisis, lack of new people involved, underestimation of difficulties, absence of complete autonomous local staff team, absence of economic support from the government. The solutions suggested have been: need of a complete local team; creation of new partnerships with NGOs, Charity Associations, Foundations, Scientific Societies; need for a bigger “Fund-Raising” plan in the US and EU; need for new and younger people involved at every level in the project.

## Conclusions

Despite the great scientific progress in the medical care of cardiovascular disease in the western countries, a significant proportion of children with CHD living in developing countries do not receive proper diagnosis and treatment.

The solution to this problem, especially in Sub-Saharian African area, will still require a long-time and it can only be done through better programming, collaboration and economic contribution of the public and private sectors.

International authorities (scientific societies and private medical companies) should identify and support the few active cardiac centers in order to finance them and concentrate both economic and human resources efforts. To do this, we need to open an international debate and create a productive cooperation between the associations already present in this sector sharing experiences and resources.

## Author contributions

All authors listed have made a substantial, direct and intellectual contribution to the work, and approved it for publication.

### Conflict of interest statement

The authors declare that the research was conducted in the absence of any commercial or financial relationships that could be construed as a potential conflict of interest.
